# Trapped between food, heat, and insects: Movement of moose (*Alces alces*) and exposure to flies in the boreal forest of Alaska

**DOI:** 10.1002/ece3.11625

**Published:** 2024-06-22

**Authors:** Bridgett M. Benedict, Daniel P. Thompson, John A. Crouse, Gabriel L. Hamer, Perry S. Barboza

**Affiliations:** ^1^ Department of Ecology and Conservation Biology Texas A&M University College Station Texas USA; ^2^ Alaska Department of Fish and Game Kenai Moose Research Center Soldotna Alaska USA; ^3^ Department of Entomology Texas A&M University College Station Texas USA; ^4^ Department of Rangelands Wildlife and Fisheries Management Texas A&M University College Station Texas USA

**Keywords:** *Alces alces*, Diptera, flies, moose, movement

## Abstract

Moose (*Alces alces*) in the boreal forest habitats of Alaska are unlike other northern ungulates because they tolerate high densities of flies (Diptera) even though flies cause wounds and infections during the warm summer months. Moose move to find food and to find relief from overheating (hyperthermia) but do they avoid flies? We used GPS collars to measure the rate of movement (m⋅h^−1^) and the time spent (min⋅day^−1^) by enclosed moose in four habitats: wetlands, black spruce, early seral boreal forest, and late seral boreal forest. Fly traps were used in each habitat to quantify spatio‐temporal abundance. Average daily air temperatures increased into July when peak biomass of forage for moose was greatest in early seral boreal forest habitats (424.46 vs. 25.15 kg⋅ha^−1^ on average in the other habitats). Average daily air temperatures were 1.7°C cooler in black spruce than other habitats, but fly abundance was greatest in black spruce (approximately 4‐fold greater on average than the other habitats). Moose increased their movement rate with counts of biting flies (mosquitoes, black flies, horse and deer flies), but not non‐biting flies (coprophagous flies). However, as air temperature increased (above 14.7°C) moose spent more time in fly‐abundant black spruce, than early seral boreal forest, showing great tolerance for mosquitoes. Warm summer temperatures appear to cause moose to trade‐off foraging in fly‐sparse habitats for resting and dissipating heat in shady, wet habitats with abundant flies that adversely affect the fitness of moose.

## Graphical abstract

Warm summer temperatures cause moose to trade‐off foraging in fly sparse‐habitats for resting and dissipating heat in shady, wet habitats preferred by flies that adversely affect the fitness of moose. (Created with BioRender.com).

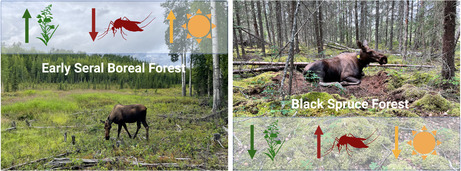

## 1 INTRODUCTION

The effects of people, wolves, and bears on moose populations are well studied (Boutin, 1992), but much less is known about the effects of flies (Diptera) that can alter both behavior and physiology of ungulates (Samuel et al., 2001). The skin and coat is the first line of defense against flies; breaks in this barrier, either from injury or molt, leave the animal vulnerable to flies (Benedict & Barboza, 2022). Fly contact or bites can result in allergic reactions, blood loss, secondary infection, restricted breathing, pneumonia, peritonitis, and neurological impairments, all of which can decrease body condition to reduce birth rates and increase death rates in a population (Ezenwa, 2004; Samuel et al., 2001).

As fly exposure increases, many ungulates react with behavioral avoidance (Benedict & Barboza, 2022). In the presence of flies, bison (*Bison bison*) trade‐off foraging for wallowing, grooming, and standing (McMillan et al., 2000; Meagher, 1973; Melton et al., 1989). Similarly, caribou (*Rangifer tarandus*) have been observed standing more in the presence of some fly species such as tabanids and oestrids (Mörschel & Klein, 1997; Raponi et al., 2018). Caribou also escape flies by moving to exposed ridges and higher elevations with cold winds and ice, which increases energy expended on movement and reduces time for feeding on high quality forage (Hagemoen & Reimers, 2002; Mörschel & Klein, 1997; Weladji et al., 2003). Fly harassment of ungulates coincides with the highest demands for lactation when females must spend most of their time foraging (Cook et al., 2021; Shively et al., 2019).

Moose appear less reactive and more tolerant to flies than caribou and bison (Benedict & Barboza, 2022). Moose do not make large‐scale movements to evade flies even though an individual moose may be surrounded by thousands of flies at any one time in the summer (Benedict et al., 2024; Benedict & Barboza, 2022). However, we do not know if moose make small scale movements and habitat choices in response to flies, or a particular type of fly. North American moose even have their own obligate species of fly, the moose fly (Muscidae: *Haematobosca alcis*); a biting species that completes its entire life cycle on or around moose (Benedict et al., 2024; Lankester & Sein, 1986). The amount of time moose spend in different habitats and microclimates is affected by environmental variables (e.g., temperature and humidity), predation, and habitat attributes such as canopy cover, understory composition, and water (Thompson et al., 2021; Timmermann & McNicol, 1988; Verzuh et al., 2022). Movement of moose in summer is influenced by foraging and the effects of warm temperatures, radiant heat loads, and metabolic heat from movement and metabolism (Thompson et al., 2021). Moose spend the majority of daylight hours (68%) in the summer bedded (Herberg, 2017; Verzuh et al., 2022) where both shade and wet soils allow cooling, and provide cover from predators (Jennewein et al., 2020; Verzuh et al., 2022). Radiant heat loads, wind, and fly activity all change the heart rate of bedded female moose (Renecker & Hudson, 1990). Movement rates are greatest in the morning when moose forage in early seral boreal forest (Thompson et al., 2021). Some groups of flies may cause moose to move faster and further to seek habitat attributes that reduce exposure to flies (Renecker & Hudson, 1990; Thompson et al., 2021), however, this has never been rigorously tested. Moose calves and adults exhibit signs of annoyance with flies, especially large horse and deer flies, by shaking their head, blowing their nose, running, jumping, twitching, stomping, scratching, and trying to nudge flies off with their nose (Benedict et al., 2024; Benedict, Thompson, et al., 2023). However, neither calves nor adults show elevated levels of glucocorticoid hormones, showing that even though flies affect behavior, flies do not cause a physiological stress response in moose (Benedict et al., 2024; Benedict, Thompson, et al., 2023).

During the summer, adult moose shed their winter coat for thermoregulation; concurrently fly abundances increase, allowing biting flies to penetrate their thin coat (Benedict, Barboza, et al., 2023). As molt occurs, round sores with severe eosinophilic and ulcerative dermatitis have been seen to progressively appear on the hind legs above the tibio‐tarsal joint, on moose in North America (Benedict, Barboza, et al., 2023; Lankester & Samuel, 2007; Murie, 1934). The sores are likely caused by legworm (*Onchocerca* sp.), carried by black flies (Diptera: Simuliidae) (Benedict, Barboza, et al., 2023). The sores leave the moose exposed to secondary infections and the cost of tissue repair and immune response. Moose with more sores have lower concentration of serum albumin probably because body protein is used for wound repair (Benedict et al., 2024). Moose in better body condition have more sores, which suggests that tolerating flies, trading‐off increased exposure for forage, may allow individual moose to attain high intakes of energy and protein to offset the costs of repairing wounds from flies (Benedict et al., 2024; Shively et al., 2019).

Exposure to flies is affected by season, habitat, and weather conditions including ambient temperature, wind, relative humidity, precipitation, light, and cloud cover (Rogy et al., 2019; Russell et al., 1993; Weladji et al., 2003). Fly life cycles are often complex because development includes multiple stages, which for some species includes both aquatic and terrestrial habitats (Culler et al., 2018). Environmental conditions do not uniformly affect all species of flies to the same extent; Anderson and Nilssen (1998) found more horse and deer flies (Tabanidae) in the morning and more mosquitoes (Culicidae) in the evening and overnight, as temperature decreased, in a tundra‐like biome of northern Norway. Shipp et al. (1987) found that the energy and water balance of a black fly (*Simulium arcticum*) was correlated to vapor pressure, air temperature, light intensity, and wind gust velocity in a prairie of central Alberta, Canada. Many flies have an upper and lower limit for activity; Russell et al. (1993) did not catch any mosquitoes below 7°C or at wind speeds above 6 m⋅s^−1^ in the northern Yukon, Canada.

We used adult female moose habituated to people at the Kenai Moose Research Center to study daily movements in relation to the forage, temperature, and number of flies in four habitats: wetland, black spruce (*Picea mariana*) forest, early seral boreal forest, and late boreal seral forest (e.g., Section 2.2). Our goal was to understand small scale moose movements (movement rates) and habitat choices (time spent in each habitat) in response to flies, forage, and temperature, exploring trade‐offs among the habitats and variables. We measured the amount of available forage in habitats at peak biomass in July (Shively et al., 2019). Environmental conditions of temperature were monitored in each habitat along with the counts of flies to develop predictors of fly abundance.

We first quantify and compare forage, ambient air temperature, and flies in the four habitats. We then model the movement rates of moose through the season and day, to understand bouts of foraging and resting without the complexity of flies. Then we model and analyze the effects of forage, ambient air temperature, and flies on the movement rates of moose. We end our analyses by modeling the effects of season, followed by ambient air temperature and flies on the amount of time moose spend in the four habitats. First, we predicted that moose movement rates would increase with higher counts of flies, thus avoiding flies and minimizing their risk to wounds and infections from flies. Second, we predicted that moose would spend most of their time in habitats with high forage abundance but that high ambient temperatures would increase the use of closed canopy habitats with shade from the sun (i.e., less radiant heat) and wet habitats with more heat dissipation (i.e., conductive heat loss). Third, we predicted that moose would avoid flies by decreasing their time spent in habitats with high abundances of flies.

## 2 MATERIALS AND METHODS

### 2.1 Study system

This study was conducted at the Kenai Moose Research Center operated by the Alaska Department of Fish and Game on the Kenai National Wildlife Refuge (60°43′ N, 150°26′ W), a boreal forest area in south central Alaska, USA (Appendix 1). All procedures for care, handling, and experimentation of animals were approved by the Animal Care and Use Committee, Alaska Department of Fish and Game, Division of Wildlife Conservation (IACUC protocol no.0086) and by the Institutional Animal Care and Use Committee, Texas A&M AgriLife Research (AUP 2019‐009A and 2021‐009A). Tame adult (2–19 y old) female moose (2019: *n* = 11; 2021: *n* = 12), some of which gave birth (2019: *n* = 5; 2021: *n* = 5) and were lactating (2019: *n* = 3) throughout the summer, were able to freely roam two 2.6 km^2^ outdoor enclosures. Moose had access to water and natural forage habitats; mixed seral state boreal forest, black spruce forest, wetlands, open meadows, and lakes (e.g., Section 2.2).

### 2.2 Vegetation

Six habitats and a lake make up the Kenai Moose Research Center; 2% wetland (kettle ponds and/or sphagnum peat bogs with areas of standing water), 3% water, 6% black spruce forest (stands dominated by black spruce), 21% early seral boreal forest (mixed deciduous forest previously disturbed by mechanical clearing 2–5 years ago and in early growth with an open canopy), 24% mid seral boreal forest (~25 years post‐disturbance), 34% late boreal seral forest (65+ years post‐disturbance), and 9% open meadow (Thompson et al., 2021; Appendix 1). Vegetation was sampled on July 28 and 29, 2022, in all six habitats. We used double weight sampling (Coulloudon et al., 1999) to estimate available biomass of the dominant summer moose forages identified by fecal analysis (Shively et al., 2019). We estimated biomass of fireweed (*Chamerion angustifolium*), and the leaves of rose (*Rosa acicularis*), highbush cranberry (*Viburnum edule*), shrub birch (*Betula glandulosa*), Alaska birch (*Betula neoalaskana*), aspen (*Populus tremuloides*), Scouler's willow (*Salix scouleriana*), and Bebb willow (*Salix bebbiana*).

Weight units for each vegetation species was established by simulated “moose bites” from observations of captive moose within the Kenai Moose Research, comparable to simulated diets collected for caribou (Denryter et al., 2022; Thompson & Crouse, 2024). Three 50‐m transects were randomly conducted in five of the habitats, and six transects were conducted in the early seral boreal forest due to the variability of the habitat. At 0 and 50 m of each transect, tree biomass surveys were conducted in a 5.64‐m radius circle (100 m^2^). The number of forage trees by species were counted; with forage trees being defined as any tree that is >1.37 m tall, and either <5 cm diameter at breast height (DBH, moose can break over trees >3 m tall and < 5 cm DBH), or any available forage <3 m off the ground (branches from trees >5 cm DBH remain within reach of a moose). “Moose bites” (sections where leaves would be stripped by a moose from the woody stems) on up to 10 of the nearest trees to point center of each forage tree species were counted and DBH was recorded. At every 5 m from 0 to 45 m, along each transect, 1 m^2^ plots were evaluated for “moose bites.” All available “moose bites” for each forage species of herbaceous, shrub, and tree seedlings (<1.37 m tall) that originate within the plot were counted. Representative “moose bite” samples were collected on odd number transects in each habitat, with additional new species collected on even number transects. Number of “moose bites” collected ranged from 10 to 40 depending on the size of the species (e.g., 10 bites for large paper birch leaves and 40 bites for small dwarf birch leaves). Vegetation samples were collected into plastic bags, frozen, and later dried to constant mass with a freeze dryer to determine moisture content and dry mass per bite (Thompson & Barboza, 2014). Dry mass of moose forage was calculated for each vegetation species by multiplying the average dry mass per bite by the total number of moose bites per area, and then summing across vegetation species to get total dry mass of moose forage (vegetation) per area for each habitat (dry mass kg·ha^−1^).

### 2.3 Flies

In 2019 we collected flies from 12 sites 18 times between May 21 and August 17 across three black spruce forests, three early seral boreal forests, three late seral boreal forest, and three wetlands for a total of 216 collections, covering 51 days (Appendix 1). Flies were also collected 50 times from one open meadow site, containing a National Oceanic and Atmospheric Administration (NOAA) weather station. From May 21 to August 14 of 2021, flies were collected 14 times from the same three black spruce sites and one open meadow site used in 2019 to capture between year differences from the most fly abundant sites, for a total of 56 collections. The locations of the sites were chosen to represent the physiographic range of each type of habitat in the study area. Trapping at each of the 13 sites consisted of one CO_2_‐baited (dry ice) Center for Disease Control and Prevention miniature light trap with ultraviolet light (John W. Hock Company, Gainesville, FL, USA) and one sticky trap (Knight Stick Biting Fly Trap, BugJammer, Inc., Pennington, NJ, USA) set with their bases at approximately 1.3 m above the ground for approximately 24 h (one trap‐day; 23.82–24.24 h). Flies were killed by either acetone exposure or citrus adhesive remover (Goo Gone, Gurnee, IL, USA), and stored frozen for analysis. Flies were transported under a USDA Veterinary Permit (139420 Research). Flies were identified morphologically and counted under a dissection microscope into the following seven groups: biting muscid flies (Muscidae), coprophagous flies (various families), mosquitoes (Culicidae), black flies (Simuliidae), horse and deer flies (Tabanidae), biting midges (Ceratopogonidae), and snipe flies (Rhagionidae). Functional groups of flies were chosen for consistency and comparability with previous studies of these species on moose (Benedict et al., 2024; Benedict & Barboza, 2022; Benedict, Thompson, et al., 2023). Coprophagous flies are the only non‐biting group of fly collected; all others are biting flies.

### 2.4 Environmental conditions

Weather was recorded in conjunction with fly sampling in 2019: one HOBO Pro V2 temperature and relative humidity data logger and one HOBO pendant temperature and light data logger (Onset, Bourne, MA, USA) were set at all 13 of the trap sites across five habitat types (i.e., wetland, black spruce, early seral and late seral boreal forest, and open meadow at the weather station). Loggers were installed at approximately 1.2 m high on steel T‐posts. In addition, at each habitat type, one operative temperature logger was installed to better capture the thermal environment the animal experiences as operative temperature incorporates ambient air temperature, radiant temperature, and air movement (Olson et al., 2014). Operative temperature loggers consisted of a HOBO water temperature Pro V2 data logger (Onset, Bourne, MA, USA) installed in a black globe, hung 0.75 m above ground and 15 cm from the trunk on the northeast side of a tree (Olson et al., 2014). In 2021, we recorded ambient air temperature at the same 13 trap sites with the addition of two sites in mid‐seral boreal forest and two sites in open meadow habitats. We also added one temperature logger within a black globe at each of the five habitat types to record operative temperature that included the effect of radiant heat load. Loggers recorded ambient air temperature (°C) and operative temperature (°C) every 5–15 min. The NOAA US Climate Reference Network weather station (AK Kenai 29 ENE; Diamond et al., 2013) which recorded ambient air temperature at 5‐min intervals, and was used to validate our HOBO loggers.

### 2.5 GPS collar deployment

We deployed Vertex plus‐4 GPS collars with 15 min fixes (Vectronics, Berlin, Germany) on moose during May 2021 while animals were immobilized for other research (Benedict, Barboza, et al., 2023). Collars had a 99.99% success rate, erroneous fixes were disregarded.

### 2.6 Calculations and statistics

In order to answer our questions about the daily movements of moose we first quantified and compared the forage, ambient air temperature, and flies in four habitats that represent the heterogeneity of the study area. Before directly incorporating these variables into movement rate and time spent models for moose, we first understood how moose use these four habitats throughout the season and day. These analyses helped us understand bouts of foraging and resting, without the complexity of flies. Models that included flies used daily averages to align with daily counts of flies.

Statistical comparisons were performed in STATA version 16.0 (StataCorp, 2019) and R Statistical Software version 4.3.1 (R Core Team, 2023), using packages “lmerTest” (Kunzetsova et al., 2017), “MuMIn” (Barton, 2010), and “nlme” (Lindstrom & Bates, 1990). We performed model selection on all mixed and logistic regression models using Akaike's information criterion (AIC*c*), selecting for the most parsimonious model (i.e., with the fewest parameters) among those with substantial support (ΔAICc < 2) (Burnham & Anderson, 2002). All variables used are presented in Table 1, continuous variables were scaled between 0 and 1 prior to analysis. A correlation matrix was conducted for each model's predictor variables in order to test for collinearity among variables (|*r*| > .7, Appendix 2; Dormann et al., 2013), correlated predictor variables were not used in the final models. Vegetation values (forage biomass) distinctly represent habitat, and thus the nominal habitat variable was replaced by vegetation values to conduct correlation matrices.

**TABLE 1:** Definitions of variables used to assess the abundance of flies and movement of moose at the Kenai Moose Research Center, Kenai Peninsula, Alaska, USA.

Variables	Units	Definition
Flies	ln flies·24 h^−1^	Daily fly counts, in aggregate, from all groups
Mosquitoes	ln mosquitoes ·24 h^−1^	Daily mosquitoes counts
Black flies	ln black flies·24 h^−1^	Daily black fly counts
Horse & Deer flies	ln horse & deer flies·24 h^−1^	Daily horse & deer fly counts
Coprophagous flies	ln coprophagous flies·24 h^−1^	Daily coprophagous fly counts
Ordinal date (date)	Days	Ordinal Date
Time	24 h	Time of day
Year	Category	Year
Habitat	Category	Habitat
Vegetation (veg)	Dry mass kg·ha^−1^	Average dry mass of moose bites per habitat in July
Ambient air temperature (Ta)	°C	Average daily ambient air temperature of a habitat recorded by HOBO loggers
Operative temperature	°C	Average daily operative temperature of a habitat recorded by HOBO loggers
NOAA ambient air temperature	°C	Average daily ambient air temperature recorded by the NOAA weather station
Time spent	min·day^−1^	Amount of time each individual moose spent in each habitat, each day
Movement rate	m·h^−1^	Rate each individual moose moved in a given hour
Average daily movement rate	m·h^−1^	Average daily rate each individual moose moved

*Note*: Abbreviations used in models shown in parenthesis.

To test whether HOBO devices accurately recoded weather variables, we used simple linear regression to analyze the relationship between HOBO and NOAA ambient air temperature at the weather station site (i.e., open meadow). HOBO ambient air temperature was highly correlated with NOAA ambient air temperature (*R* ^2^ = .926, *p* = .000; Appendix 3) with a positive slope of 1.056 ± 0.017 SE. HOBO devices consistently recorded similar values for temperature as the NOAA weather station in the open meadow, and thus HOBO measurements were used in all future analyzes within habitats. We used simple linear regression to examine the effects of ordinal date on ambient air temperature, to understand seasonal trends. Bonferroni pairwise comparisons were conducted to compare vegetation, ambient air temperature, and operative air temperature among all habitats. We focus our analysis on wetland, black spruce forest, early seral boreal forest, and late boreal seral forest as these four habitats represent the complexity of the site.

All daily fly counts were corrected to represent flies collected for 24 h (flies·24 h^−1^), totaled across the two fly trap types, and natural log corrected for normality. Bonferroni pairwise comparisons were performed comparing changes in each fly group among all habitats. Using simple linear regression models, we modeled counts of flies for each fly group using ambient air temperature, ordinal date, habitat, and the interaction between habitat and ambient air temperature, with ordinal date and ambient air temperature as both linear and quadrics to allow for seasonal variation and upper and lower temperature thresholds (Russell et al., 1993, 2013). The top model for each fly group was then used to predict counts of flies at every GPS collar location for each moose.

The locations of moose by habitat were determined by overlaying moose GPS collar locations with vegetation polygons (ArcMap 10.6.1; ESRI, Redland, CA, USA) (Thompson et al., 2021) to calculate the amount of time each individual moose spent in each habitat, each day. Euclidean distance was calculated for each successive GPS location to calculate movement rate of each moose, and then averaged per day per moose to calculate daily movement rate (m·h^−1^) of each moose.

We then used mixed‐effects regression to describe the effects of age of the moose, season (ordinal date), and time of day on movement rate of moose, with random intercepts and coefficients on ordinal date by individual moose to account for repeated measures of dependent variables. Ordinal date included a quadratic term and time of day included a quadratic, cubic, and quartic term to allow for seasonal variation and daily fluctuations in movement (Herberg, 2017; Thompson et al., 2021). To further analyze movement rate we used mixed‐effects regression to examine the effects of ambient air temperature, vegetation, and each fly group on average daily movement rate, with random effects of individual moose to account for repeated measures of dependent variables.

We used mixed‐effects regression to describe time spent by moose in each habitat, each day for wetlands, black spruce, early seral and late seral boreal forest. The first model included ordinal date, with individual moose as random effects. The second model included ambient air temperature and each fly group, with individual moose as random effects.

## 3 RESULTS

### 3.1 Habitat

**FIGURE 1:**
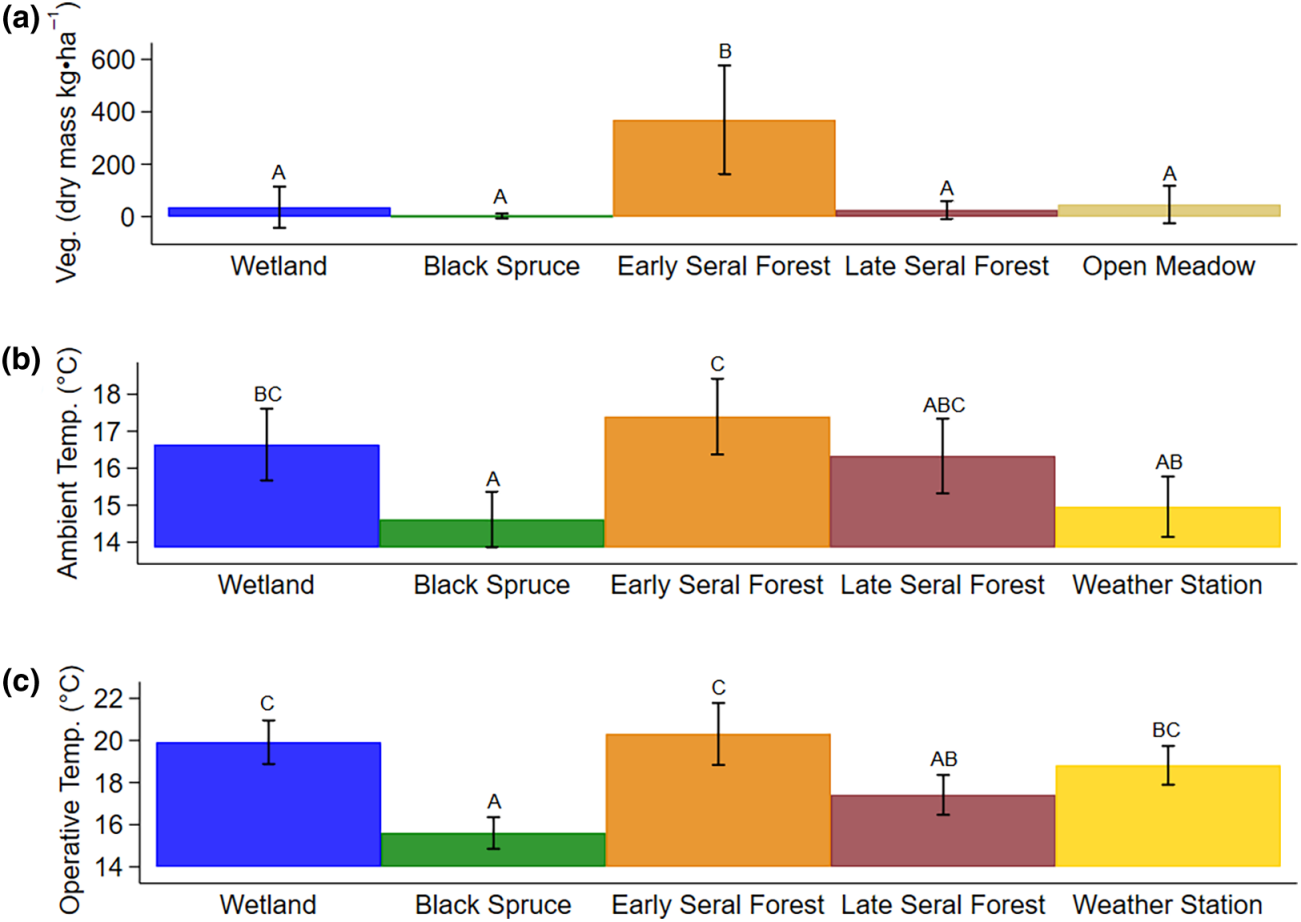
Average dry mass of vegetation (kg·ha^−1^; a), average daily ambient air temperature (°C; b), and average daily operative temperature (°C; c) in wetland, black spruce, early seral boreal forest, late seral boreal forest, and open meadow habitats at the Kenai Moose Research Center, Kenai Peninsula, Alaska, USA. Vegetation was measured in open meadow at multiple sites, but temperature was measured only at the NOAA weather station for the open meadow habitat in 2019. Error bars indicate 95% confidence intervals around the estimate. In each panel, habitat classes with the same letter indicate no significant difference between means (*p* < .05).

July moose forage biomass varied from a minima of 2.2 kg·ha^−1^ black spruce to a maxima of 424.46 kg·ha^−1^ in early seral boreal forest (Figure 1a). Average daily ambient air and operative temperature also varied by habitat, with warmer temperatures in early seral boreal forests and cooler temperatures in black spruce forests (Figure 1b,c). Furthermore, air temperatures increased with ordinal date (*R* ^2^ = .308, *p* = .000).

### 3.2 Flies

A total of 102,812 flies were trapped, identified, and assigned to seven functional groups. We collected 88.0% of all flies in CO_2_ baited light traps, with the remainder in sticky traps (Appendix 4). CO_2_ baited light traps were more effective in capturing biting midges (99.9%), mosquitoes (94.5%), biting muscid flies (93.8%), and black flies (67.2%; Appendix 4a–c,f). Sticky traps were more effective in capturing snipe flies (61.6%), and horse and deer flies (72.0%; Appendix 4e,g). Trap success varied within taxa, by habitat. For coprophagous flies, CO_2_ baited light traps were more effective in black spruce, but both traps were similarly effective in other habitats (Appendix 4d).

**FIGURE 2:**
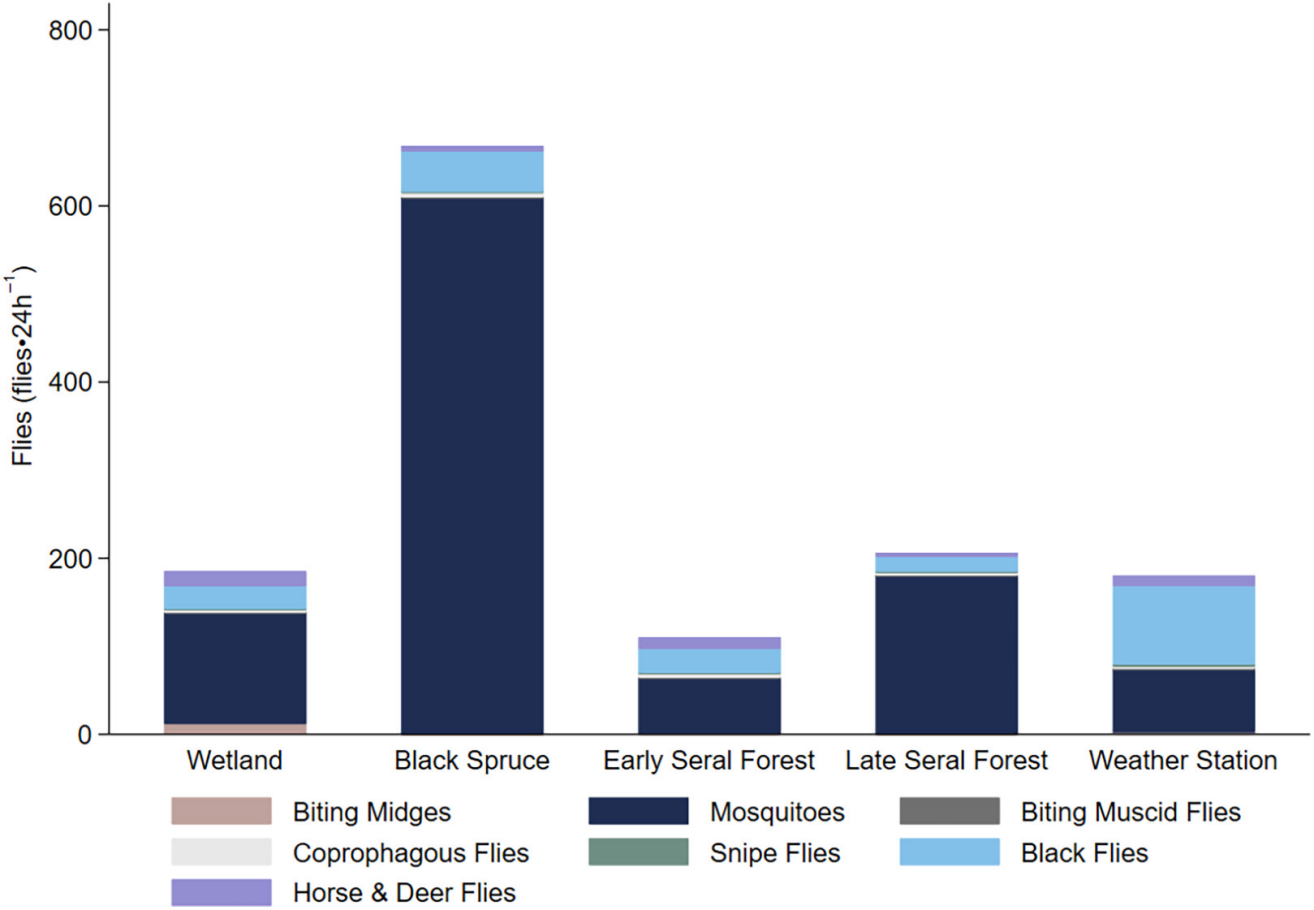
Average flies collected (flies·24 h^−1^) from both traps (sticky trap and CO_2_ baited light trap) combined in wetland, black spruce, early seral boreal forest, late seral boreal forest, and weather station open meadow habitats, with fly groups stacked by color, at the Kenai Moose Research Center, Kenai Peninsula, Alaska, USA (Appendix 5).

The majority of flies collected from both traps at each site were mosquitoes (80.8%) followed by black flies (13.5%), horse and deer flies (3.2%; Figure 2). Less than 3% of remaining flies were coprophagous flies (1.3%), biting midges (0.8%), snipe flies (0.3%), and biting muscid flies (0.03%; Figure 2). We collected an average of 320 flies per site in each 24 h trap period (Figure 2). Flies collected varied from a maximum of 49.5% (668 flies⋅24 h^−1^) from black spruce habitats to a minimum of 8.2% (110 flies⋅24 h^−1^) from early seral boreal forest habitats (Figure 2). Counts of mosquitoes, coprophagous flies, and black flies in black spruce were significantly different than in other habitats (Figure 2, Appendix 5). Similarly, counts of coprophagous flies and black flies at the weather station were significantly different than in other habitats (Figure 2, Appendix 5).

**FIGURE 3:**
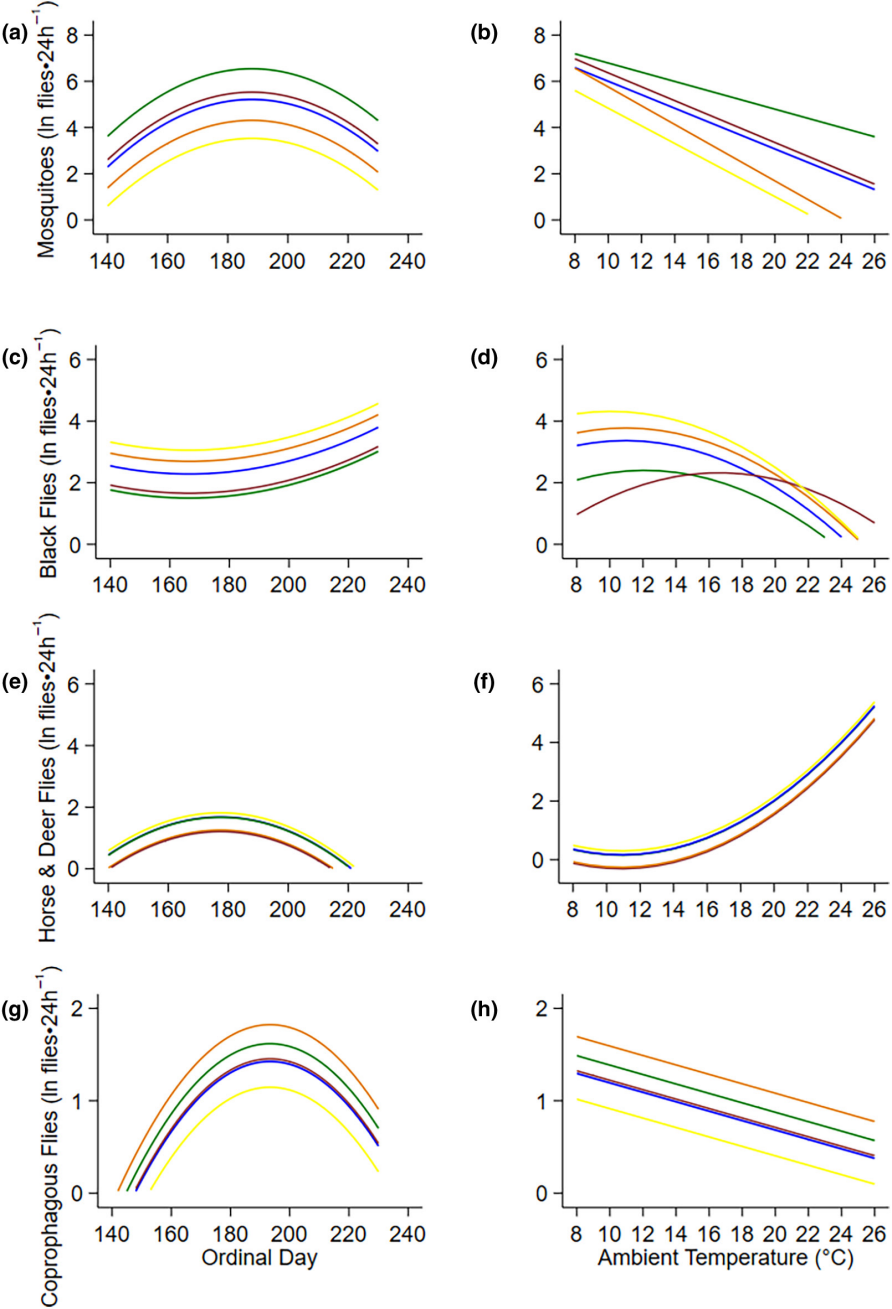
Relationships between rate of capture of flies (ln flies·24 h^−1^) and environmental conditions (ordinal date, ambient air temperature (°C)). Lines are marginal predictions from linear regression models (Table 2) for mosquitoes (ln flies·24 h^−1^; a, b), black flies (ln flies·24 h^−1^; c, d), horse & deer flies (ln flies·24 h^−1^; e, f), and coprophagous flies (ln flies·24 h^−1^; g, h) in each habitat (wetland = blue, black spruce = green, early seral boreal forest = orange, late seral boreal forest = maroon, weather station = yellow) at the Kenai Moose Research Center, Kenai Peninsula, Alaska, USA.

The top models that described fly abundance, mosquitoes, black flies, coprophagous flies, horse and deer flies included ambient air temperature, ordinal date, and habitat (Table 2, Figure 3, Appendix 2A). Mosquitoes and black flies additionally included an interaction between habitat and ambient air temperature (Table 2). Sample sizes were not sufficient for models to appropriately converge on a solution for biting muscid flies, biting midges and snipe fly groups, and thus they were left out of further analysis (Table 2). Counts of mosquitoes, horse and deer flies, and coprophagous flies rose to a peak at ordinal date 188 (July 7; Figure 3a), 177 (June 26; Figure 3b), 193 (July 12; Figure 3g) respectively, and then declined slowly through summer. In contrast, counts of black flies were lowest at ordinal date 166 (June 15) and rose through summer (Figure 3c). Abundances of mosquitoes and coprophagous flies decreased with increasing daily air temperatures (Figure 3b,h), while black fly abundances increased to 12°C before decreasing with increasing temperatures (Figure 3d). On the contrary, horse and deer flies increased with increasing temperatures beginning at 12°C (Figure 3f).

**TABLE 2:** Summary of best top simple linear regression model from each set of candidate models (in the top 2 AICc lowest units) for flies at the Kenai Moose Research Center, Kenai Peninsula, Alaska, USA.

Response	Model	ΔAIC_c_	*ω*	*k*	Deviance	*R* ^2^
Flies	Ta + date + date^2^ + habitat	1.83	0.25	10	−434.99	.51
Mosquitoes	Ta + date + date^2^ + habitat + habitat:Ta	0.55	0.33	14	−207.26	.71
Black flies	Ta + Ta^2^ + date + date^2^ + habitat + habitat:Ta	1.58	0.13	15	−189.25	.36
Biting midges	Habitat + date + date^2^	0.70	0.09	9	−547.46	.13
Biting muscid flies	Date + date^2^	1.21	0.14	5	−641.57	.06
Coprophagous flies	Ta + date + date^2^ + habitat	0.00	0.33	10	−136.09	.24
Snipe flies	Ta + date + date^2^ + habitat	1.17	0.17	10	−485.97	.08
Horse and deer flies	Ta + Ta^2^ + date + date^2^ + habitat	0.89	0.23	11	−268.32	.63

*Note*: All models include one additional parameter as the associated error term.

Abbreviations: Deviance, measure of model fit; *k*, number of estimable parameters; *R* ^2^, coefficient of determination; Ta, ambient air temperature; ΔAIC, difference between model AIC and lowest AIC in the model set; ω, akaike model weight.

### 3.3 Behavior of moose

**FIGURE 4:**
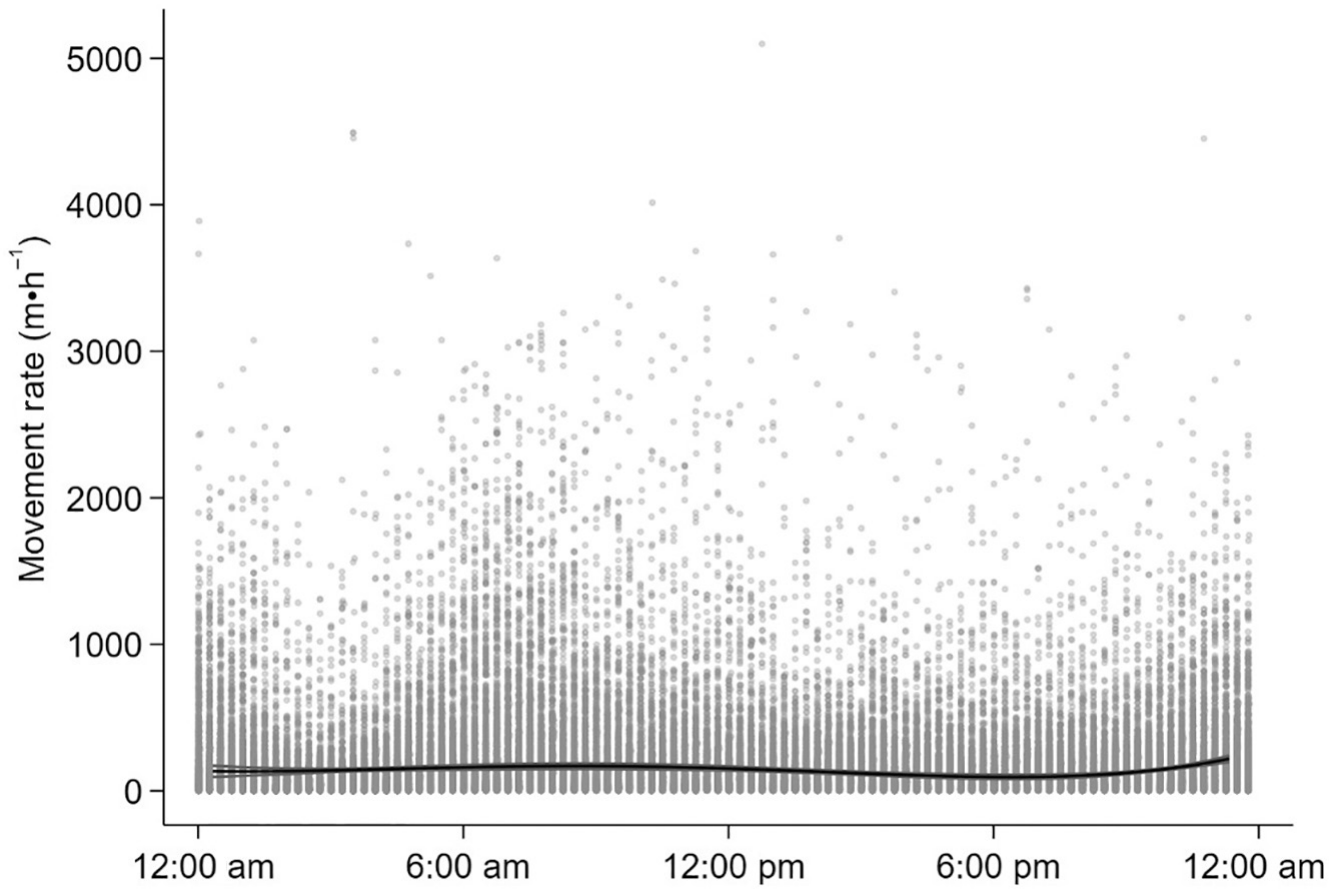
Relationships between movement rate (m·h^−1^) and time of day for moose (*n* = 12) at the Kenai Moose Research Center, Kenai Peninsula, Alaska, USA. Dots are observations and lines are marginal predictions with 95% confidence intervals from mixed‐effect regression models (Table 3).

Movement rate (m·h^−1^) was correlated with age, ordinal date and time of day (Table 3, Figure 4, Appendix 2B). On average, older moose moved less in each hour than younger moose over the summer. Average movement rates declined by 38.52 m·h^−1^ as summer progressed. Movement rates were greatest at 8:30 am and 11:00 pm, and slowest at 6:00 pm (Figure 4).

**TABLE 3:** Summary of best top mixed‐effects regression model from each set of candidate models (in the top 2 AICc lowest units) for movement rate and time captive adult female moose spent in selected habitats at the Kenai Moose Research Center, Kenai Peninsula, Alaska, USA.

Response	Model	ΔAIC_c_	ω	*k*	Deviance
Movement rate*	age + date + date^2^ + time + time^2^ + time^3^ + time^4^	1.44	0.29	10	−310,138
Average daily movement rate**	veg + mosquitoes + black flies + coprophagous flies + horse and deer flies	1.91	0.26	9	−2489.34
Time spent in early seral boreal forest**	date + date^2^	0.00	1.00	6	−937.00
	Ta + mosquitoes + black flies + coprophagous flies + horse and deer flies	0.00	1.00	9	−1673.28
Time spent in late seral boreal forest**	date + date^2^	0.00	0.53	6	−446.38
	mosquitoes + black flies + coprophagous flies + horse & deer flies	0.00	0.62	8	−1687.57
Time spent in Black spruce forest**	date + date^2^	0.00	0.75	6	−1234.34
	Ta + mosquitoes + black flies + coprophagous flies	0.00	0.51	8	−1899.31
Time spent in wetlands**	(date not in top model)	0.00	0.36	4	−1124.31
	Ta + mosquitoes + black flies + coprophagous flies + horse & deer flies	0.00	0.66	9	−1214.29

Abbreviations: Deviance, measure of model fit; *k*, number of estimable parameters; Ta, ambient air temperature; ΔAIC, difference between model AIC and lowest AIC in the model set; ω, Akaike model weight.

*Random intercepts and coefficients on ordinal date by individual moose to account for repeated measures of dependent variables, and the associated error term. **Random intercept of individual moose to account for repeated measures of dependent variables, and the associated error term.

**FIGURE 5:**
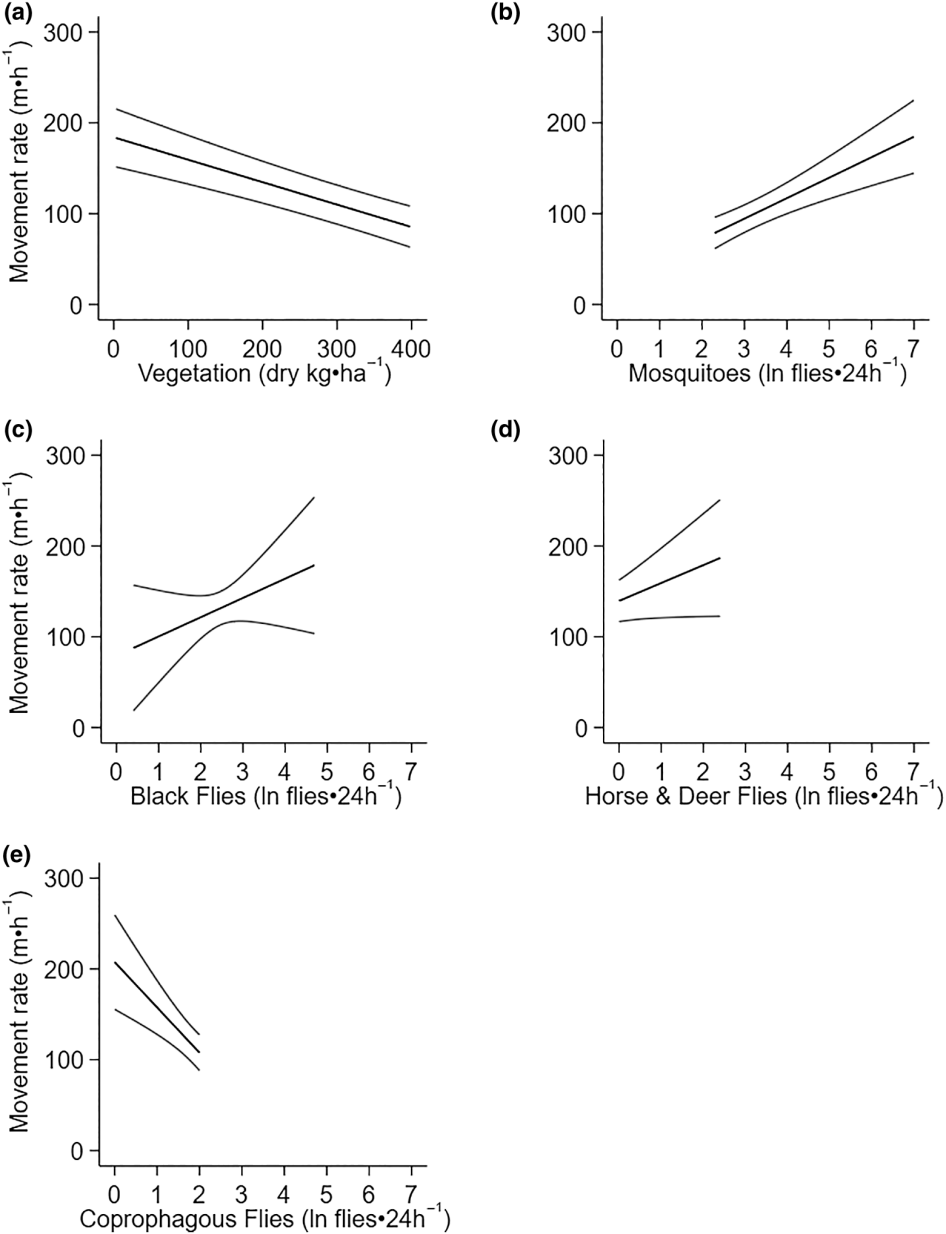
Relationships between average daily movement rate (m·h^−1^) of moose (*n* = 12) and exposure to summer moose forage biomass (kg·ha^−1^; a) and predicted mosquitoes (ln flies·24 h^−1^; b), black flies (ln flies·24 h^−1^; c), horse and deer flies (ln flies·24 h^−1^; d), and coprophagous flies (ln flies·24 h^−1^; e) at the Kenai Moose Research Center, Kenai Peninsula, Alaska, USA. Lines are marginal predictions with 95% confidence intervals from mixed‐effect regression models (Table 3).

Average daily movement rate was related to the average daily summer moose forage biomass and average daily biting (mosquitoes, black flies, horse and deer flies) and non‐biting (coprophagous) flies (Table 3, Figure 5, Appendices 2C and 6). Moose moved more slowly in habitats with higher biomass of summer forage (Figure 5a). Moose moved faster (78.71 vs. 184.77 m·h^−1^) as mosquito abundance increased (2.3 vs. 7 ln flies⋅24 h^−1^) (Figure 5b). They also moved faster as the other two biting fly groups increased; black fly, horse and deer flies (Figure 5c,d), but not as non‐biting fly abundance increased (Figure 5e).

**FIGURE 6:**
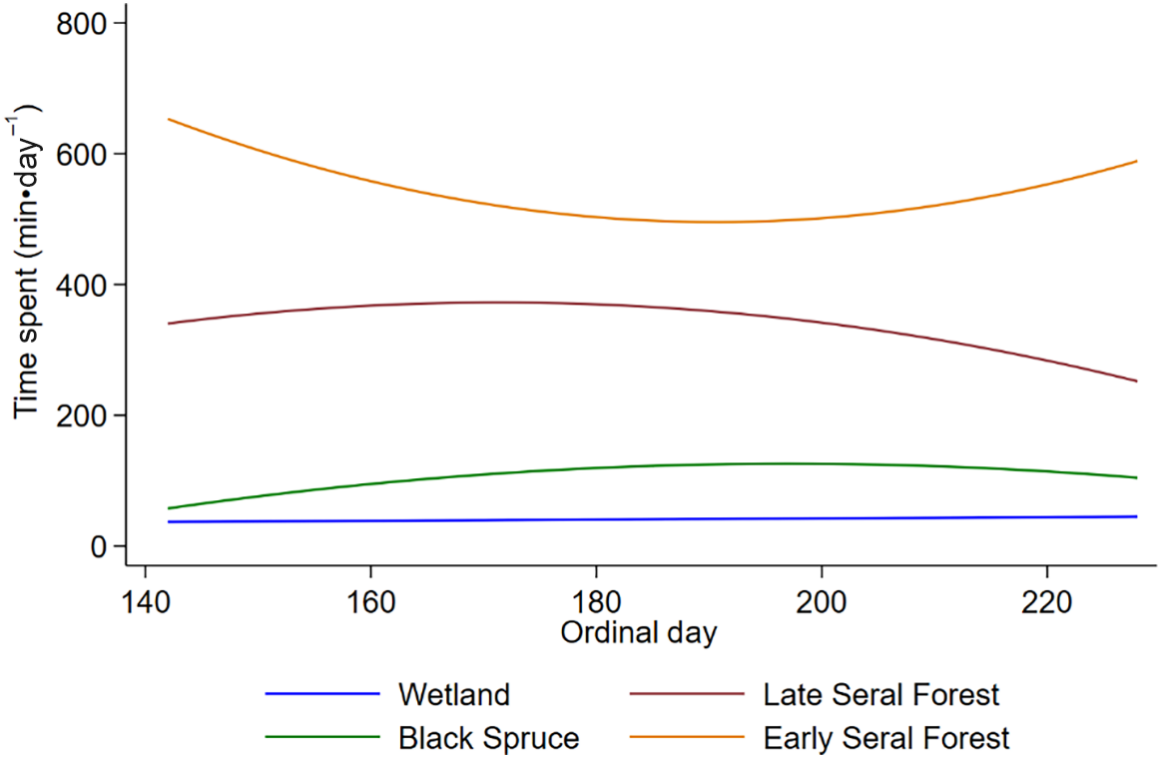
Relationships between daily time spent by moose (*n* = 12, min·day^−1^) in each habitat and ordinal date. Lines are marginal predictions from mixed‐effect regression models (Table 3) for wetland, black spruce, early seral boreal forest, and late seral boreal forest at the Kenai Moose Research Center, Kenai Peninsula, Alaska, USA.

**FIGURE 7:**
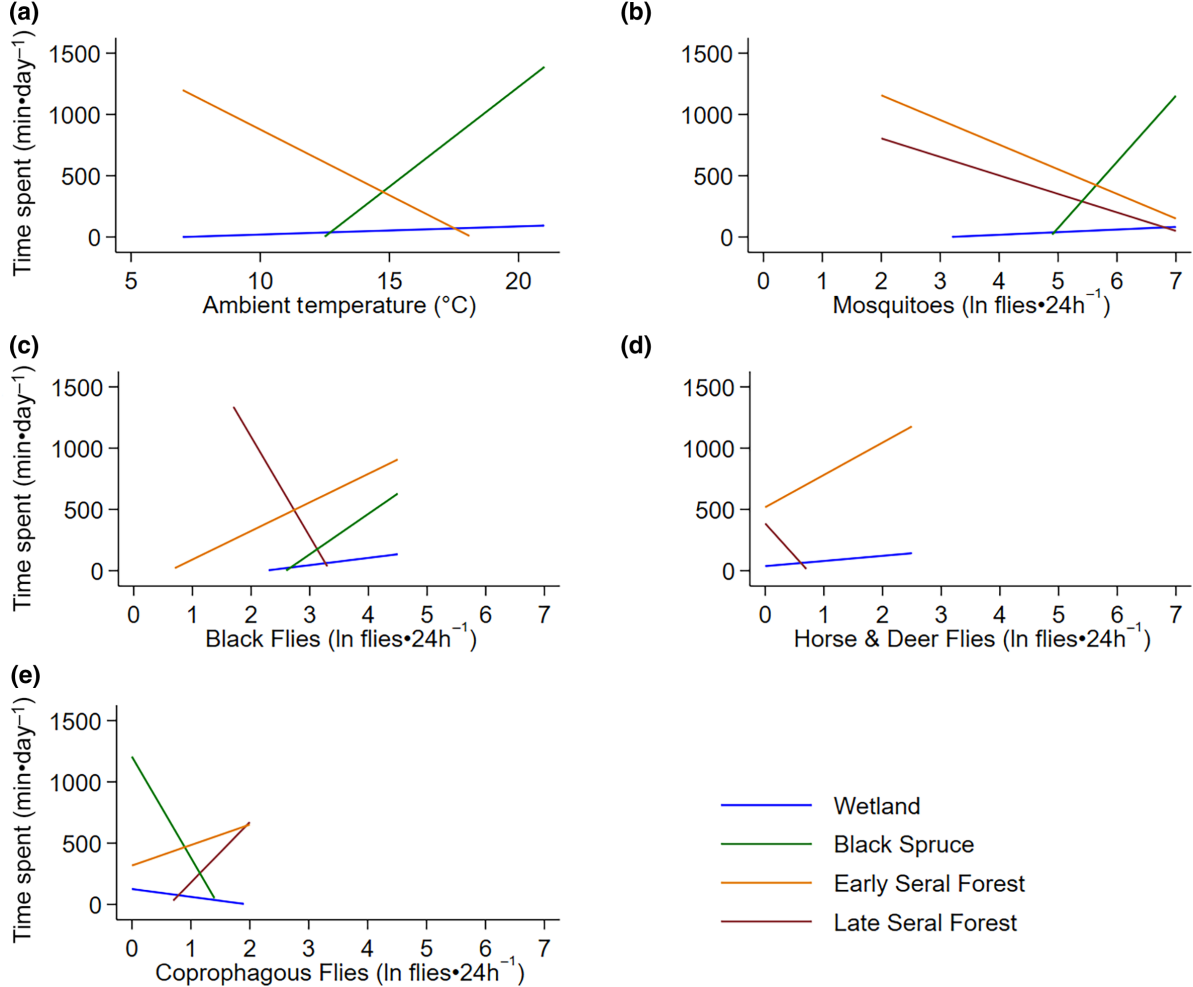
Relationships between daily time spent by moose (*n* = 12, min·day^−1^) in each habitat and ambient air temperature (°C; a), and predicted mosquitoes (ln flies·24 h^−1^; b), black flies (ln flies·24 h^−1^; c), horse & deer flies (ln flies·24 h^−1^; d), and coprophagous flies (ln flies·24 h^−1^; e). Lines are marginal predictions from mixed‐effect regression models (Table 3) for wetland, black spruce, early seral boreal forest and late seral boreal forest at the Kenai Moose Research Center, Kenai Peninsula, Alaska, USA.

Moose spent most of their time in early seral boreal forests, followed by late seral boreal forests, black spruce, and then wetland across the summer (Figure 6). The amount of time moose spent in early seral boreal forests and wetlands was related to average daily ambient air temperature and exposure to biting and non‐biting flies, while time spent in late seral boreal forests was not related to air temperature (Table 3, Figure 7, Appendix 2D and 6). The amount of time moose spent in black spruce was only related to ambient air temperature and abundances of mosquitoes, black flies, and coprophagous flies (Table 3, Figure 7, Appendix 2D and 6). As air temperature increased moose spend less time in early seral boreal forests and more time in black spruce forests, surpassing time spent in early seral at 14.8°C (Figure 7a). Moose spent less time in early seral boreal forests with increasing abundances of mosquitoes, but more time with increasing abundances of black flies, coprophagous flies, horse and deer flies (Figure 7b–e). They spent more time in black spruce and wetlands with increasing abundances of mosquitoes and black flies, but less time with increasing coprophagous flies (Figure 7b,c,e). They also spent more time in wetlands with increasing counts of horse and deer flies (Figure 7d). Although effects of temperature and fly exposure were significant in wetlands, those effects were much smaller than predicted for other habitats (Figure 7). As counts of biting flies increased in late seral forest moose spent less time in this habitat, but the opposite relationship was seen with coprophagous flies (Figure 7b–e).

## 4 DISCUSSION

As predicted, moose spent the majority of their time in the forage abundant, and fly sparse early seral boreal forests (Figure 6), moving to forage in the morning and resting in the evening around 6:00 pm (Table 3, Figure 4). Movement rate did increase with exposure to flies as predicted, but only from biting flies (mosquitoes, black flies, horse and deer flies) and not non‐biting flies (coprophagous flies) (Figure 5). However, as air temperature increased moose spent more time in fly abundant black spruce, than early seral boreal forest (Figure 7a). Moose trade off foraging in less fly abundant habitats for cooling in shaded moist black spruce habitats that reduce radiant heat loads and allow conductive heat loss (Figure 7). Moose tolerated flies, particularly mosquitoes, in exchange for cooling (Figure 7).

### 4.1 Foraging

Foraging accounts for most of the movement of moose as spring ambient air temperatures rise, and both growing plants and flies emerge (Renecker & Hudson, 1986; Shively et al., 2019; Thompson et al., 2021). High movement rates in the morning and at night, and low movement rates in the evening (Figure 4) probably reflect daily patterns of foraging and resting (Table 3, Figure 5a) that coincide with rising and declining body temperatures in moose (Thompson et al., 2021). Moose spent most of their time in early seral boreal forests (Figure 6), moving the slowest (Figure 5a) in this forage abundant habitat (Figure 1a). The benefits of foraging in this food‐rich habitat probably diminished as flies became abundant (Figure 3) and ambient air temperatures rose from spring to summer.

### 4.2 Behavioral thermoregulation

As ambient temperatures increased moose spent significantly less time in early seral boreal forest and more time resting under canopy in black spruce habitats, surpassing time spent in early seral at 14.8°C (Figure 7a) (Thompson et al., 2021). In comparison to early seral boreal forest, black spruce provides less forage but more shade from radiant heat loads and moist bedding sites that allow conductive heat loss (Figure 1) (Alston et al., 2020; Olson et al., 2016; Van Cleve et al., 1983). Mosses insulate the soil, reducing soil temperatures, and contribute to the formation of permafrost beneath black spruce forests (Oechel & Van Cleve, 1986; Van Cleve et al., 1983). Thermoregulatory models of moose indicate that canopy cover can mitigate heat stress up ≤10°C ambient air temperature, but temperatures above 10°C require access to wet ground and water for conductive cooling (Alston et al., 2020; McCann et al., 2016; Verzuh et al., 2022). Increasing time spent in black spruce likely reflects cooling at rest (Figure 7). Wet ground (wetland habitats) and canopy cover (late seral boreal forest habitats) alone do not meet the needs of both requirements, like black spruce habitats.

### 4.3 Flies and temperature

Moose likely do not make large scale movements to escape flies, in general they speed up when there are high abundances of biting flies (Figure 5) but they must tolerate flies in trade‐off for other resources (Figure 7). When heat gained while foraging is dissipated at rest in shady, wet black spruce habitats preferred by flies (49.5% of flies were collected in black spruce; Figure 2), the fitness of moose is adversely affected (Benedict et al., 2024). This trap of preferring the most fly abundant habitat, with little to no forage, for cooling will likely worsen as climate change progresses (Mallory & Boyce, 2018; Thompson et al., 2021). There is a need for future studies to study the diel activity of fly groups in association with moose diel activity to further parse out the finer details of fly‐moose relationships, particularly for the less abundant groups of flies trapped. This study should also be conducted with wild, truly free‐ranging moose which exist at lower densities (Herreman, 2022) and may differ in their reactions.

### 4.4 Implications and conclusions

Heat loads and flies impact a moose's ability to maximize forage consumption in a complex and constantly changing environment through summer. Moose miss opportunities to forage in summer when they seek relief in cool shady habitats with less forage. Moose, especially in the north temperate zone of the boreal forest, already have a narrow summer window for capturing a short period of growth of vegetation through high intakes of browse (Renecker & Hudson, 1986; Shively et al., 2019). Summer and spring temperatures, particularly late spring, create a foraging window in which moose must assimilate enough protein and energy before winter when forage abundance and quality are low. Moose are capital breeders; calving rates are dependent on a female's body condition at the time of rut, in the fall (Allen et al., 2017). What is gained in the spring and summer affects their health, fecundity, survival, and the survival of their calves throughout the year (Allen et al., 2017).

The costs of tolerating flies may extend into winter because flies can expose moose to parasites. We recently described the effect of filarial nematodes on moose in the Kenai Peninsula, Alaska (Benedict et al., 2024; Benedict, Barboza, et al., 2023; Benedict, Thompson, et al., 2023). Legworm (*Onchocerca* sp.), likely transmitted by black flies, likely causes the open sores on the legs of adult moose and led to a decrease in serum protein (Benedict et al., 2024; Benedict, Barboza, et al., 2023), while *Setaria yehi*, likely transmitted by mosquitoes, led to morbidity and mortality in calves (Benedict, Thompson, et al., 2023). Both mosquitoes and black flies are tolerated by moose and make up the majority of flies in the boreal forests of the Kenai Peninsula, creating repeated spring and summer exposure. Nematode parasites have also been found to cause neurological impairments, peritonitis, and death in a declining moose population in northern Minnesota (Grunenwald et al., 2016, 2018; Murray et al., 2006).

Moose residing in warming regions at the southern end of their distribution may be forced to trade off fly relief for thermal refuge. Moose populations are constrained by predators, heat stress, and parasites in the southern range, but the northern range has expanded historically and continues to expand (Monteith et al., 2015; Murray et al., 2006; Tape et al., 2016). Since 1850, climate warming has facilitated the growth of riparian shrubs and earlier snowmelt to provide foraging corridors for moose to expand from the boreal forest into the arctic slope (Tape et al., 2016). Flies are however abundant in the tundra and likely to limit foraging gains at the northern limits of the distribution.

Boreal moose need abundant black spruce and early seral boreal forest in summer to cope with the effects of flies and heat stress. They need black spruce with moist, wet understories for cooling and early seral boreal forests for browsing. They must maintain high levels of browsing to offset periods of lost foraging opportunities and for tissue repair, and to maintain high fecundities that replace cohorts of calves lost to predators and disease. Fire is the primary driver of succession in boreal forests, creating valuable early seral boreal forest with increased forage quantity and quality (Brown et al., 2018; Davis & Franzmann, 1979). Black spruce habitat is historically resilient to fire, having a high flammability, but depending on fire for regeneration and replacing itself quickly after being burned (Baltzer et al., 2021). Drier climatic conditions and more severe fires have limited the ability of black spruce to regenerate (Baltzer et al., 2021). An outbreaks of spruce bark beetle (*Dendroctonus rufipennis*) on the Kenai Peninsula in the 1990s also increased fire and fire severity by creating surface fuels and killing the less resilient white spruce (*Picea glauca*) (Hansen et al., 2016). A careful balance of fire severity is important for maintaining moose habitat and thus populations of boreal moose in Alaska for wildlife and human communities.

## AUTHOR CONTRIBUTIONS

**Bridgett M. Benedict:** Conceptualization (lead); data curation (lead); formal analysis (lead); funding acquisition (supporting); investigation (lead); methodology (lead); project administration (lead); resources (equal); supervision (supporting); validation (lead); visualization (lead); writing – original draft (lead); writing – review and editing (lead). **Daniel P. Thompson:** Conceptualization (supporting); data curation (supporting); formal analysis (supporting); investigation (supporting); methodology (supporting); project administration (supporting); resources (supporting); supervision (supporting); validation (supporting); visualization (supporting); writing – review and editing (supporting). **John A. Crouse:** Conceptualization (supporting); data curation (supporting); funding acquisition (equal); investigation (supporting); methodology (supporting); project administration (supporting); resources (equal); supervision (equal); visualization (supporting); writing – review and editing (supporting). **Gabriel L. Hamer:** Conceptualization (supporting); formal analysis (supporting); methodology (supporting); resources (equal); visualization (supporting); writing – review and editing (supporting). **Perry S. Barboza:** Conceptualization (equal); data curation (supporting); formal analysis (equal); funding acquisition (lead); investigation (supporting); methodology (supporting); project administration (lead); resources (lead); software (lead); supervision (equal); validation (equal); visualization (equal); writing – original draft (supporting); writing – review and editing (equal).

## CONFLICT OF INTEREST STATEMENT

The authors declare that they have no conflict of interest.

## Data Availability

GPS locality data for the moose in this study is available upon reasonable request from the Alaska Department of Fish and Game and subject to a data sharing agreement per Alaska Statute 16.05.815(d). All other data and code is available at https://doi.org/10.18738/T8/YMBRYG.

## APPENDIX 1

### 1.1

Kenai Moose Research Center study area map with habitats and sampling location defined.

## APPENDIX 2

### 2.1

A. Correlation matrix of predictor variables (ordinal date (date), ambient air temperature (Ta), habitat represented by vegetation forage biomass (habitat)) used in model of fly abundance.

**Table:**

	Date	Ta	Habitat
Date	1.0000		
Ta	0.5506	1.0000	
Habitat	0.0105	0.2038	1.0000

B. Correlation matrix of predictor variables (ordinal date (date), time of day (time), and age of moose (age)) used in model of movement rates of moose.

**Table:**

	Date	Age	Time
Date	1.0000		
Age	0.0000	1.0000	
Time	−0.0077	0.0000	1.0000

C. Correlation matrix of predictor variables (flies and vegetation (veg)) used in model of average daily movement rates of moose.

**Table:**

	Veg	Mosquitoes	Black flies	Coprophagous flies	Horse and deer flies
Veg	1.0000				
Mosquitoes	−0.1251	1.0000			
Black flies	0.4866	−0.3519	1.0000		
Coprophagous flies	−0.0109	0.5348	0.1248	1.0000	
Horse and deer flies	−0.2313	0.2489	−0.7104	0.3099	1.0000

D. Correlation matrix of predictor variables (ambient air temperature (Ta) and flies) used in model of time moose spent in habitats.

**Table:**

	Temp	Mosquitoes	Black flies	Coprophagous flies	Horse and deer Flies
Temp	1.0000				
Mosquitoes	−0.4852	1.0000			
Black flies	−0.0890	−0.3519	1.0000		
Coprophagous Flies	0.2342	0.5348	0.1248	1.0000	
Horse and deer flies	0.4636	0.2489	−0.7104	0.3099	1.0000

## APPENDIX 3

### 3.1

Validation of the HOBO loggers at the NOAA weather station open meadow site at the Kenai Moose Research Center, Kenai Peninsula, Alaska, USA. Linear regression between HOBO measured ambient air temperature (°C) and NOAA weather station measured ambient air temperature (°C) plotted (circles). Solid orange lines are 1:1 comparison between HOBO and NOAA measures.

## APPENDIX 4

### 4.1

Average flies (flies·24 h^−1^) collected in a sticky trap (solid bar) versus a CO_2_ baited light trap (muted bar) by fly group (a–g) at the Kenai Moose Research Center, Kenai Peninsula, Alaska, USA, with habitat collected shown in colors.

## APPENDIX 5

### 5.1

Bonferroni comparison of fly groups across habitats (wetland, black spruce, early seral boreal forest, late seral boreal forest, and weather station) at the Kenai Moose Research Center, Kenai Peninsula, Alaska, USA. For each fly group, habitats sharing the same letter have ≤5% differentiation.

**Table:**

Habitats	Fly groups						
	Biting midges	Mosquitoes	Biting Muscid flies	Coprophagous flies	Snipe flies	Black flies	Horse and deer flies
Wetland	A	A	A	AB	A	A	A
Black spruce	A	C	A	B	A	AB	A
Early boreal seral forest	A	A	A	AB	A	A	A
Late boreal seral forest	A	A	A	AB	A	A	A
Weather station	A	A	A	A	A	B	A

## APPENDIX 6

### 6.1

Beta coefficients for mosquitoes, black flies, coprophagous flies, horse and deer flies, vegetation, and ambient air temperature with standard errors from best mixed‐effects regression models for average daily movement rate model (a) and time spent models, by habitat (b) at the Kenai Moose Research Center, Kenai Peninsula, Alaska, USA.
